# Recent Medical Jurisprudence

**Published:** 1892-12

**Authors:** Henry A. Riley

**Affiliations:** New York


### RECENT MEDICAL JURISPRUDENCE.

BY HENRY A. RILEY, A. B., LL. B., New York.

### PRECOCIOUS WICKEDNESS.

THE annals of crime contain few cases so remarkable in precocious wickedness as an investigation developed a short time since in England. There were two boys, named Shearon and Crawford, aged respectively eight and nine years of age, who were arraigned before a magistrate for drowning another lad, named Eccles, who was eight years old.

Shearon and Crawford, who were vicious children, plotted together to take the clothing of the first well-dressed boy they met. They induced Eccles to go with them and take a swim. The pond where they took him was twelve feet deep in some parts, and they got a raft and paddled it to one of the deep places. They then pushed Eccles off, and although he made frantic efforts to save himself, they would not let him on the raft again for a long time. After a while they pulled him up, and, towing the raft to where it was a little deeper, pushed him off again. He pleaded hard for his life, but they had no mercy, and soon Eccles' strength gave out and he sank under the water. The two boys then conveyed the body to the shore and beat it until they were sure it was dead.

After watching it a considerable time, and seeing no movement, they divided Eccles' clothing and went home. When the boys were arrested shortly afterward, they confessed all the horrible details, but without apparent feeling.

So extraordinary a case seems to baffle all criminal procedure, and to make uncertain what course to pursue. It does not seem possible that boys of such a tender age could, under any circumstances, be executed for murder, but the wickedness of the crime could hardly be exceeded by men of mature age.

### ARE HYPODERMIC INJECTIONS OF MORPHINE LAWFUL?

A suit has just been begun, in South Dakota, by a Mrs. C. M. Sweitzer against a doctor living in Aberdeen. She alleges that the doctor repeatedly "pumped morphine into her husband so that he became a morphine fiend," and being intellectually and morally a wreck, was unable to provide for her support. The medico-legal questions involved in this suit are of considerable interest, as what is called the morphine habit is becoming one of the notable medical signs of the day. The facility with which hypodermic injections can be made, and their possible results when long continued, make it the duty of the physician to be careful when he adopts such a line of treatment.

Some of the newspapers are clamoring for a law placing some restrictions upon the administration of morphine, alleging that it is as dangerous as the sale of liquors to minors. Whether the consent of parents or husbands is necessary to make a hypodermic injection of morphine proper, is a question which has never been before a court for decision, but if heavy doses were given and continued for a considerable length of time, without informing the patient of the character of the treatment, and evil results followed, it is very probable that the physician would be liable in damages.

### BLOOD POISONING AND CASES OF ASSAULT.

Some curious medico-legal points were recently developed in an ordinary assault and battery case in New York City.

The wife of a professional light-weight boxer and pugilist, who was dignified by the title of "Professor Haley," was accosted at night by a man in the streets, who followed her to her home. She tried to get away from him, but he went into the house directly after her.

She then called up-stairs to her husband, who came down quickly to chastise the intruder. He took to his heels, but the athletic training of the Professor enabled him to catch up with the fleeing man, and in the scuffle which followed, the stranger was struck in the mouth and two of his teeth knocked out.

The Professor's knuckles were badly cut as the result of the blow, but he paid no attention to them until they began to swell and the arm to grow painful.

The physician who was called in pronounced the case one of blood poisoning, and, although everything possible was done for the sufferer, he became worse, and it was decided that amputation could not even save him. Death occurred in about a week. The offender was not arrested, and no clue was found, when the case was decided to be one of blood poisoning.

### LEGISLATION FOR THE PROTECTION OF THE EYE.

The Maine Legislature, at the last session, passed the following law:

SECTION 1. Should one or both eyes of an infant become reddened or inflamed at any time within four weeks after its birth, it shall be the duty of the midwife, nurse, or person having charge of said infant, to report the condition of the eyes at once to some legally qualified practitioner of medicine of the city, town, or district, in which the parents of the child reside.

SEC. 2. Any failure to comply with the provision of this Act shall be punishable by a fine not to exceed one hundred dollars, or imprisonment not to exceed six months, or both.

This law was modeled after a similar law placed on the statute book in New York in 1890, and was, no doubt, prompted by the fact that blindness or serious disease of the eyes dates, in many cases, from birth, and could be avoided by prompt medical treatment. Legislation of the same character will be proposed in other States at the next session of the Legislature.

### THE DEFECTS OF OUR IMMIGRATION LAWS.

The number of immigrants to this country does not seem to decrease under the workings of the new Federal law, as only a trifling number can be actually shown to be suffering from insanity, or dangerous contagious diseases, and there are many ways of temporarily placing enough money in the hands of the shiftless emigrant, so that he will not seem to be necessarily a public burden. The public sentiment of the country is certainly crystallizing in favor of more stringent laws on the subject, and the visit of Mr. Weber, the chief of the Immigration Bureau at New York, to the principal ports of embarkation in Europe, will be likely to furnish valuable information in regard to the best methods of restriction.

The most effectual remedy would probably be a charge of $25 for each steerage passenger landed, but this would be sure to cause bitter opposition on the part of a large number of foreign-born persons now in this country. The tendency of immigration toward this land is strikingly illustrated by the reports just issued by the English customs department. This shows that of 69,087 pauper immigrants who landed at British ports during the first six months of this year, 53,177 were described as being *en route* to the United States.—*North American Practitioner.*

